# The pitfalls of rDNA‐based AMF identification: a comparative analysis of rDNA and protein‐coding genes

**DOI:** 10.1111/nph.70557

**Published:** 2025-09-12

**Authors:** Franck Stefani, Mario Laterrière, Lobna Abdellatif, Claudia Banchini, Greta Mader Stevens, Sylvie Séguin, Kasia Dadej, Lisa Koziol, Wendy Findlay, Yolande Dalpé

**Affiliations:** ^1^ Ottawa Research and Development Centre Agriculture and Agri‐Food Canada Ottawa ON K1A 0C6 Canada; ^2^ Québec Research and Development Centre Agriculture and Agri‐Food Canada Quebec QC G1V 2J3 Canada

**Keywords:** arbuscular mycorrhizal fungi, DNA barcoding, intragenomic polymorphism, PacBio sequencing, protein‐coding genes, rDNA copies, species delimitation

## Abstract

- Intragenomic polymorphism of rDNA in arbuscular mycorrhizal fungi (AMF) has been largely overlooked in ecological and taxonomic studies, and the reliability of nuclear rDNA regions for species identification has not been comprehensively evaluated and compared with protein‐coding genes.
- Analysis of rDNA copies from *Rhizophagus irregularis* strains revealed average intragenomic distances ranging from 0.08% (small subunit) to 6.9% (internal transcribed spacer 2 (ITS2)), with a maximum of 21.1% in ITS1 within strain DAOM 197198. Intragenomic rDNA polymorphisms are widespread throughout the AM fungal phylogeny, as confirmed by single nucleotide polymorphism density analysis and PacBio sequencing of 148 AM fungal cultures representing 44 species. All commonly targeted rDNA loci in ecological and taxonomic studies are polymorphic, with ITS and large subunit being the most variable, leading to paraphyletic clades and misleading phylogenetic interpretations among closely related species.
- No unique genetic distance threshold can be applied to identify AMF, because none of the examined protein‐coding genes or partial rDNA had a barcode gap. However, indicative distance thresholds of 1% for glomalin, 1.1% for RPB1, and 1.7% for H^+^‐ATPase provide guidance for species delimitation.
- This study characterizes the extent of intragenomic rDNA polymorphism in AMF, underscores the taxonomic challenges posed by highly variable loci, and describes a bioinformatics pipeline for recovering rDNA copies.

## Introduction

Since the first DNA sequences were obtained from arbuscular mycorrhizal fungi (AMF, Glomeromycota) (Simon *et al*., 1992), several loci of the nuclear ribosomal operon (rDNA) and a few protein‐coding genes have been sequenced and evaluated for their usefulness for the delimitation and identification of AM fungal species. However, intragenomic variation of rDNA, the shortage of universal AMF primers to amplify protein‐coding genes, the release of sequences from misidentified cultures in public repositories, and the lack of comparative analyses to assess the performance of the various DNA loci available make the discovery and identification of AM fungal species problematic.

Arbuscular mycorrhizal fungi are identified either by sequencing one or multiple consecutive loci along the nuclear rDNA using high‐throughput sequencing in environmental studies (e.g. Berruti *et al*., 2017; Rodríguez‐Echeverría *et al*., 2017; Delavaux *et al*., 2021; Kolaříková *et al*., 2021; Malicka *et al*., 2022; Metzler *et al*., 2024), or by a polyphasic approach combining spore morphological analyses together with Sanger sequencing of a 1.5‐kb fragment of the rDNA and protein‐coding gene in taxonomic studies (e.g. Krüger *et al*., 2009, 2012; Stockinger *et al*., 2010; Błaszkowski *et al*., 2022, 2024; Niezgoda *et al*., 2024). Among the protein‐coding genes used to identify AMF, the largest subunit of RNA polymerase II (RPB1) (Stockinger *et al*., 2014) is most frequently sequenced in studies describing new AM fungal species, but other protein‐coding genes are occasionally included, such as elongation factor 1‐α, H^+^‐ATPase (Sokolski *et al*., 2010), phosphate transporter gene (Sokolski *et al*., 2011), and glomalin (Magurno *et al*., 2019). Overall, rDNA is still preferred over protein‐coding genes for profiling AM fungal communities and identifying AM fungi, thanks to primer sets that work for all Glomeromycota lineages (e.g. Lee *et al*., 2008; Krüger *et al*., 2009) and multiple copies within the genome, which facilitates amplification and sequencing.

However, whole‐genome sequencing of five strains related to the model AM fungal species *Rhizophagus irregularis* revealed unusual rDNA features within Glomeromycota that are not seen in other fungal lineages. In most fungi, the rDNA operon is organized in tandem repeats of tens to hundreds of copies (Rooney & Ward, 2005; Lofgren *et al*., 2019), which are usually subject to concerted evolution (Eickbush & Eickbush, 2007; Naidoo *et al*., 2013). However, strains of *R. irregularis* only possess 9–11 divergent copies of the complete rDNA operon, distributed across four to six chromosomes (Maeda *et al*., 2018; Yildirir *et al*., 2022). The intragenomic variation among rDNA copies and its influence on precise species identification remain largely unexplored.

Environmental studies investigating AMF in soil and root samples using high‐throughput sequencing of rDNA loci downplay intragenomic variation and its potential impact on diversity metrics, while relying on untested distance thresholds as proxies for species richness. These studies typically target a short amplicon (*c*. 0.5 kb) from the rDNA region, either the small subunit (SSU) or large subunit (LSU), which are often preferred over internal transcribed spacers (ITS1 and ITS2) because of the substantial variability in ITS regions, a phenomenon recognized since the mid‐1990s (Lloyd‐Macgilp *et al*., 1996) and occasionally revisited in more recent studies using amplicon cloning and Sanger sequencing (Thiéry *et al*., 2012, 2016). While the profiling of AM fungal communities relies on these short SSU or LSU amplicons, taxonomists focus primarily on a *c*. 1.5‐kb fragment spanning from the end of the SSU to the D1 and D2 variable domains of the LSU (commonly referred to as the ‘Krüger fragment’), often in combination with a protein‐coding gene, such as RPB1. This divergence in targeted regions, together with poorly characterized intragenomic variation of rDNA loci, interferes with reliable taxonomic identification using SSU and LSU datasets and obscures species richness estimation in environmental studies.

To address this issue, a handful of studies sequenced the SSU‐ITS‐LSU region with PacBio Sequel single‐molecule real‐time (SMRT) technology to profile AM fungal communities (Schlaeppi *et al*., 2016; Symanczik *et al*., 2017; Bender *et al*., 2019; Dirks & Jackson, 2020; Kolaříková *et al*., 2021). This has the advantage of linking the SSU V4‐V5 and LSU D1‐D2 variable regions, which are otherwise targeted separately in environmental studies, while also including the ‘Krüger fragment’ for robust taxonomic assignment. However, all of these studies used arbitrary sequence similarity thresholds of 97–100% to infer operational taxonomic units (OTUs) or amplicon single variants (ASVs) from long reads, despite a lack of evidence that these thresholds effectively discriminate between intragenomic, intraspecific, and interspecific variability across the highly genetically diverse families of AMF.

What is the extent of intragenomic rDNA polymorphism among AMF? How does this variation affect the accuracy of species identification? Could protein‐coding genes provide a more reliable alternative to rDNA? To answer these questions, the intragenomic polymorphism of the nuclear rDNA (number of copies, pairwise distances, and single nucleotide polymorphisms (SNP) density) was analyzed using high‐quality whole‐genome data from Yildirir *et al*. (2022) and newly generated rDNA amplicon sequences, both from *in vitro* cultures. In addition, the effectiveness of PacBio high‐fidelity circular consensus sequencing (HiFi) reads to capture the intragenomic variability of the rDNA was evaluated in 41 AM fungal species, and a bioinformatics pipeline was developed to process the HiFi reads. Finally, the glomalin, H^+^‐ATPase and RPB1 protein‐coding genes were sequenced for 143 AM fungal cultures representing 39 species. The presence of a barcode gap, that is nonoverlapping distance distribution between the maximum intraspecific distances and minimum interspecific distances, was assessed for these three protein‐coding genes and the rDNA. Overall, this study aimed to address the challenges and limitations related to DNA‐based identification of AMF in environmental and taxonomic studies.

## Materials and Methods

### Biological material

For the overall study, spores for total genomic DNA (gDNA) extraction, amplification, and sequencing were isolated from 163 *in vivo* and *in vitro* cultures, representing 44 species from five orders and seven families. All the cultures are maintained at the Canadian Collection of Arbuscular Mycorrhizal Fungi (CCAMF). The *in vitro* cultures were established as described by Goh *et al*. (2022) and incubated in the dark at 22°C. The culture medium of the *in vitro* cultures was solubilized with citrate buffer (Doner & Bécard, 1991) to release the spores. For the *in vivo* cultures, spores were isolated by wet sieving of the pot substrate following the method of Gerdemann & Nicolson (1963). Details of the cultures used in this study are provided in Supporting Information Tables S1–S3. The cultures were identified at the species level by morphological spore analysis, multigene phylogenetic reconstruction (Table S4), and NCBI Blast search (Table S5; Altschul *et al*., 1990; Johnson *et al*., 2008).

### DNA isolation, amplification, and library preparation for SNP analysis

The distribution and frequency of SNPs in the SSU‐ITS‐LSU regions were analyzed in 16 pure *in vitro* cultures representing nine species among the families Glomeraceae *sensu* Piroz. & Dalpé (1989) and Entrophosporaceae (Table S1). *In vitro* cultures were selected to ensure the reliability of SNP analysis as they provide clonal, contaminant‐free material, allowing unambiguous attribution of SNP variation to intragenomic polymorphism. After releasing hyphae and spores from the culture medium, the fungal material was ground with a pestle in 1.5‐ml microcentrifuge tubes. Celite was added to facilitate the grinding process. Total gDNA was then isolated using the QIAamp® DNA Mini kit (Qiagen, Mississauga, ON, Canada) following the manufacturer's protocol with the following modifications: 200‐μl ATL buffer was used instead of 180‐ and 8‐μl RNAse enzyme mix was added. Samples were incubated overnight at 56°C and shaken at 900 rpm for 15 s every 30 min using an Eppendorf® ThermoMixer® (Eppendorf, Mississauga, ON, Canada). Final elution was performed in 35‐μl AE buffer. A 3.4‐kb fragment was amplified using the NS1 (White *et al*., 1990)/LSUmAr3 (Krüger *et al*., 2009) primer set (Integrated DNA Technologies (IDT), Coralville, IA, USA) in a 25‐μl reaction mix, as follows: 2 μl gDNA, 9.5 μl PCR‐grade water, 12 μl 2× Phusion Hot Start II High‐Fidelity PCR Master Mix (Thermo Fisher Scientific, Waltham, MA, USA) containing 200 μM dNTPs, 2 mM MgCl_2_, and 2.5 units of enzyme, and 1.25 μl of each primer (500 nM final concentration per reaction). Thermocycling conditions for NS1/LSUmAr3 amplification were as follows: an initial denaturation at 98°C for 30 s, followed by 30 cycles of denaturation at 98°C for 10 s, annealing/extension at 60°C for 2 min, and a final extension step at 65°C for 5 min. The reaction was held at 10°C until further processing. The 3.4‐kb PCR fragments were normalized to 460 ng and sheared to a 550‐bp insert using a Covaris M220 instrument (Covaris®, Woburn, MA, USA). The resulting insert fragments were used as templates to construct PCR‐free libraries using the NxSeq AmpFREE Low DNA Library kit (Lucigen, Middleton, WI, USA), following the manufacturer's protocol. Individual indexed libraries were pooled in equimolar quantities and sequenced on a MiSeq instrument (Illumina, San Diego, CA, USA) using the MiSeq Reagent Nano kit v.2 500 cycles (2 × 250‐bp read length; Illumina) following the manufacturer's recommendations.

### DNA isolation for PacBio rDNA sequencing and Sanger sequencing of protein‐coding genes

Spores from *in vivo* cultures were pipetted into sterile 0.2‐ml tubes (eight‐tube strips for Precellys), containing 5 μl of distilled water. Excess water was removed, and spores were air dried. Two sterilized zirconium silica beads of 1.4 mm diameter (Fox Industries, Fairfield, CT, USA) were added to each tube, followed by the addition of 5 μl of PrepMan™ Ultra Sample Preparation Reagent (Applied Biosystems™, Thermo Fisher Scientific, Waltham, MA, USA). Spores were then homogenized using a Precellys® Evolution Homogenizer (Bertin Technologies, Rockville, MD, USA) for 10 s at 8000 rpm for two cycles. Samples were briefly centrifuged and incubated at 99°C for 10 min, followed by 2 min at room temperature. After a second quick centrifugation, the liquid containing the DNA and debris was transferred to a new eight‐tube strip without beads. Samples were centrifuged at 2038 ***g*** for 5 min, and the supernatants were transferred to a 0.5‐ml tube, diluted with 10 mM Tris–HCl (pH 8.0) based on the recovered volumes (usually *c*. 4 μl) and spore size. For single spores with diameters ranging from 30 to 200 μm, a fivefold dilution was applied, a fourfold dilution for spores < 30 μm, and a sixfold dilution for spores > 200 μm. For multiple spores, *c*. 20 spores per batch, with diameters < 200 μm, a sixfold dilution was used, while batches containing 40–80 spores were diluted eightfold. DNA extracts were stored at −20°C until further analysis.

### DNA amplification and PacBio sequencing of partial rDNA

To investigate the intragenomic polymorphism of rDNA in AM fungal species for which high‐resolution genome assemblies are lacking, gDNA isolated from *in vivo* and *in vitro* cultures was amplified and sequenced using PacBio technology. Fig. S1 shows the workflow for AMF spore sequencing and analysis of the PacBio HiFi reads. A 2.8‐kb fragment of the partial rDNA was amplified as described for the SNP density analysis with the modification that the AML1 (Lee *et al*., 2008) and wLSUmBr (Schlaeppi *et al*., 2016) primer set was used instead of the NS1/LSUmAr3 primer set. Because some of the gDNA extractions were performed on *in vivo* cultures, primers specific to AMF were required. The reverse primer LSUmBr was preferred to LSUmAr3 because it targets all AMF lineages, whereas LSUmAr3 is more specific for Glomeraceae *sensu* Piroz. & Dalpé and Entrophosporaceae (Krüger *et al*., 2009). These primers were modified (Table S6) to include universal sequences at the 5′ position in order to incorporate barcodes into the PCR amplicons using the Barcoded Universal F/R Primers Plate‐96 (part no. 101‐629‐100; Pacific Biosciences, MenloPark, CA, USA) and to prepare the SMRTbell® library as described in the procedure ‘Preparing SMRTbell® Libraries using PacBio® Barcoded Universal Primers for Multiplexing Amplicons’ (partno.: 101‐791‐800 v.02, April 2020; Pacific Biosciences). Thermocycling conditions to amplify the 2.8‐kb fragment of the partial rDNA were as follows: an initial denaturation step of 98°C for 45 s followed by 30 cycles (35 cycles for single spores) at 98°C for 30 s, 58°C for 30 s, 72°C for 3 min, and a final extension step performed at 72°C for 10 min. Samples were then held at 10°C. Amplicons were visualized by electrophoresis on a 1.5% agarose gel stained with GelRed® (1 : 10 000; Biotium Inc., Fremont, CA, USA) at 60 V for 40 min and visualized using the Gel‐Doc system (Bio‐Rad Laboratories). The thermocycling conditions to incorporate the barcodes were as follows: an initial denaturation at 98°C for 30 s, followed by 26–30 cycles of 98°C for 15 s, 64°C for 15 s, and 72°C for 2 min. A final extension was performed at 72°C for 7 min, after which the samples were held at 4°C. PCR products were visualized on 2% E‐gel 96 (Thermo Fisher Scientific). After visualization, the PCR products were purified and normalized to a concentration of 5 ng μl^−1^ using the NGS Normalization 96‐well kit (Norgen Biotek Corp., Thorold, ON, Canada) before and after multiplexing the amplicons. Then equal volumes of purified and normalized PCR products were pooled together and concentrated to 50 μl total volume using NucleoSpin Gel and PCR Clean‐up kit (Macherey‐Nagel, Düren, Nordrhein‐Westfalen, Germany) or the DNA Clean & Concentrator kit (Zymo Research, Irvine, CA, USA). Final concentration of the pool was determined using a Qubit 3 Fluorometer (Thermo Fisher Scientific). Sequencing was performed at the McGill University and Genome Québec Innovation Centre (Montréal, QC, Canada) on PacBio Sequel II system using one SMRT Cell 8M with a 15‐h movie time.

### DNA amplification and sequencing for protein‐coding genes

Nested PCR was used to amplify regions of the glomalin, H^+^‐ATPase, and RPB1 genes using primer sets designed for AM fungal species at the family or order level (Table S7) due to the lack of conserved regions across the AMF phylogeny. Unless otherwise stated, the sequencing was performed with the primer set used for the second round of PCR. The reaction mixture, thermocycling conditions, gel electrophoresis, and Sanger sequencing protocols were consistent across the targeted genes, with the exception of the annealing temperatures, which were adjusted for each primer set (Table S7). Amplifications were performed in 10 μl of reaction mix as follows: 6.82 μl PCR‐grade water, 1 μl of 10× Titanium Taq PCR Buffer (TaKaRa Bio Inc., Mountain View, CA, USA), 0.5 μl of the 2 mM dNTP's mix (100 μM of each dNTP), 0.5 μl of bovine serum albumin (Thermo Fisher Scientific) at 20 mg μl^−1^ (1 μg per reaction), 0.04 μl of each primer (80 nM per reaction), 0.1 μl of Titanium Taq Polymerase (TaKaRa Bio Inc.), and 1 μl of gDNA. Thermocycling conditions for the first and second round of PCR were as follows: an initial denaturation step of 95°C for 1 min followed by 40 cycles at 95°C for 30 s, annealing temperature (Table S7) for 45 s, 68°C for 1 min 30 s, and a final extension step performed at 68°C for 3 min. Samples were then held at 10°C. Amplicons were visualized by electrophoresis as described above. Sanger sequencing was performed using the BigDye™ Terminator v.3.1 Cycle Sequencing kit (BrilliantDye™; NimaGen Inc., Nijmegen, the Netherlands). Each reaction mixture consisted of 1 μl PCR amplicon, 8.5 μl BigDye™ sequencing mix diluted 1 : 8, and 0.5 μl sequencing primer (final concentration of 3.2 μM). For each reaction, the 1 : 8 BDT sequencing mix contained 3.75 μl of sterile filtered H_2_O (BioShop Canada Inc., Burlington, ON, Canada), 2.5 μl of 20% d‐(+)‐Trehalose (BioShop Canada Inc.), 1.75 μl of 5× sequencing buffer (Thermo Fisher Scientific), and 0.5 μl of Big Dye Terminator v.3.1 (Thermo Fisher Scientific). Thermocycling conditions for Sanger sequencing were as follows: 95°C for 3 min followed by 40 cycles at 95°C for 30 s, 50°C for 15 s, and 60°C for 2 min. Samples were then put on hold at 10°C. Sanger sequencing was performed on a 3500xl Genetic Analyzer at the Molecular Technologies Laboratory of Agriculture and Agri‐Food Canada in Ottawa (Ottawa, ON, Canada).

### Bioinformatic analyses

For SNP density analysis, primers NS1 and LSUmAr3 were trimmed using Geneious® 10.1.2 (Biomatters Ltd, Auckland, New Zealand). Reads were then paired using Usearch v.10 (Edgar, 2010), with a minimum assembled length of 220 bp and a maximum of 420 bp. Quality filtering was performed in Usearch using a maximum expected error threshold of 0.25. For each culture, the rDNA region corresponding to the NS1/LSUmAr3 primer set was initially amplified and Sanger‐sequenced using internal primers (data not shown) to generate long reference sequences. These reference sequences were then used to detect and remove chimeras using Uchime v.4.2.40 (Edgar *et al*., 2011) as implemented in Geneious and to guide the mapping and assembly of the remaining paired‐end reads using the Geneious assembler. A final visual inspection of the reads was performed to remove any chimeric reads not detected by Uchime. Mapping and SNP calling was performed on each assembly using the Find Variations/SNPs feature as implemented in Geneious. The resulting annotation table for each assembly was then imported into R, and the SNP density plot was calculated using the function ‘kpPlotDensity’ from the R package karyoploter v.1.30.0 (Gel & Serra, 2017).

For the analysis of the rDNA copies from PacBio sequencing, PacBio Sequel subreads were demultiplexed and assembled into HiFi reads using SMRT® Link v.8.0 (Pacific Biosciences). Sequences with fewer than three passes and a minimum predicted accuracy of < 0.9 were removed. A bioinformatics pipeline was developed and published on GitHub (https://github.com/AAFC‐Bioinfo‐AAC/AMF‐rDNA‐PacBio‐amplicon‐sequence‐analysis‐pipeline) to recover and analyze the rDNA copies from single or multiple spores of AM fungal cultures. The pipeline was designed to run in the Snakemake v.7.32.4 (Mölder *et al*., 2021) and Conda environments. It includes the following steps: primer removal and quality filtering using Cutadapt v.4.5 (Martin, 2011), dereplication and chimera removal using Vsearch v.2.24 (Rognes *et al*., 2016), clustering using Swarm v.3.1.4 (Mahé *et al*., 2014, 2021), taxonomic identification using Blast v.2.16.0 (Altschul *et al*., 1990) and TaxonKit v.0.17.0 (Shen & Ren, 2021), elimination of non‐Mucoromycota swarm clusters and selection of swarm clusters based on their abundance values (see Supporting Information and the GitHub repository for more details), alignment of the filtered swarm clusters using Mafft v.7.49 (Katoh *et al*., 2002; Katoh & Standley, 2013).

### Phylogenetic and DNA barcoding analyses

To perform the DNA barcoding analyses on a reliable set of species, species boundaries were confirmed for 143 *in vivo* and *in vitro* cultures using genealogical concordance phylogenetic species recognition (GCPSR; Taylor *et al*., 2000) *sensu* Dettman *et al*. (2006). Fig. S2 and Table S4 show which of the glomalin, H^+^‐ATPase, and RPB1 protein‐coding genes support the recognition of monophyletic groups under the GCPSR criterion. In total, 39 phylogenetic species were delimited, representing 15 genera and 6 families (Table S3). DNA sequences were aligned using Mafft as implemented in Geneious Prime® v.2024.0, and coding sequences were translated into amino acids to identify the first, second, and third codon positions. Introns were not included in the phylogenetic and DNA barcoding analyses. The ITS1, ITS2, 5.8S, and flanking regions of the SSU and LSU rDNA were identified and extracted using ITSx v.1.1.3 (Bengtsson‐Palme *et al*., 2013). The best‐fitting substitution models for each codon and each rDNA locus were selected using ModelTest‐NG v.0.1.7 (Darriba *et al*., 2019) via the CIPRES Science Gateway (Miller *et al*., 2010) using the Akaike information criteria corrected for small sample size (Hurvich & Tsai, 1989). Phylogenetic trees were inferred using MrBayes v.3.2.7a, as implemented in CIPRES. For each analysis, two simultaneous and independent runs were performed with four Markov Chain Monte Carlo (MCMC), running for 3 million generations for the concatenated protein‐coding genes and 7 million generations for the rDNA regions. The convergence of the MCMC chains and the effective sample size values of the parameters were evaluated using Tracer v.1.7.2 (Rambaut *et al*., 2018). A burn‐in value of 30% was applied to exclude the initial trees before calculating consensus trees with Bayesian posterior probabilities. To assess the robustness of the tree topology, an additional phylogenetic analysis of the rDNA dataset was performed using IQ‐Tree v.2.3.2 (Nguyen *et al*., 2015), as implemented in CIPRES (data not shown). Substitution models were inferred for each partition (SSU, ITS1, 5.8S, ITS2, and LSU) by IQ‐Tree to determine the best‐fitting models. Tree topology was optimized using nearest neighbor interchange and subtree pruning and regrafting strategies. Branch support was estimated using ultrafast bootstrapping based on 5000 replicates. For all phylogenies, species from the genus *Paraglomus* were selected to root the trees.

To identify the optimal distance threshold for species discrimination based on intra‐ and interspecific distances, pairwise distances were calculated using the *dist.seqs* function in Mothur v.1.48.0 (Schloss *et al*., 2009). The parameters calc = eachgap and countends = F were used to penalize each gap in the sequences without penalizing terminal gaps due to unequal sequence length. Maximum intraspecific distance and minimum interspecific distances were calculated using the R package spider v.1.4‐2 (Brown *et al*., 2012). Data visualization was performed using R v.4.4.1 (R Development Core Team, 2024) and the following packages: ape v.5.8 (Paradis & Schliep, 2019), biostrings v.2.72.1 (Pagès *et al*., 2024), dplyr v.1.1.4 (Wickham *et al*., 2023a), gdata v.3.0.0 (Warnes *et al*., 2023), ggplot2 v.3.5.1 (Wickham, 2016), htmlwidgets v.1.6.4 (Vaidyanathan *et al*., 2023), karyoploter v.1.30.0 (Gel & Serra, 2017), knitr v.1.48 (Xie, 2014, 2015, 2025), plotly v.4.10.14 (Sievert, 2020), proxy v.0.4.27 (Meyer & Buchta, 2022), pwalign v.1.0.0 (Aboyoun & Gentleman, 2024), mass v.7.3.61 (Venables & Ripley, 2002), readr v.2.1.5 (Wickham *et al*., 2023b), reshape2 v.1.4.4 (Wickham, 2007), stringr v.1.5.1 (Wickham, 2022).

## Results

### Nuclear rDNA intragenomic polymorphism

**Fig. 1:**
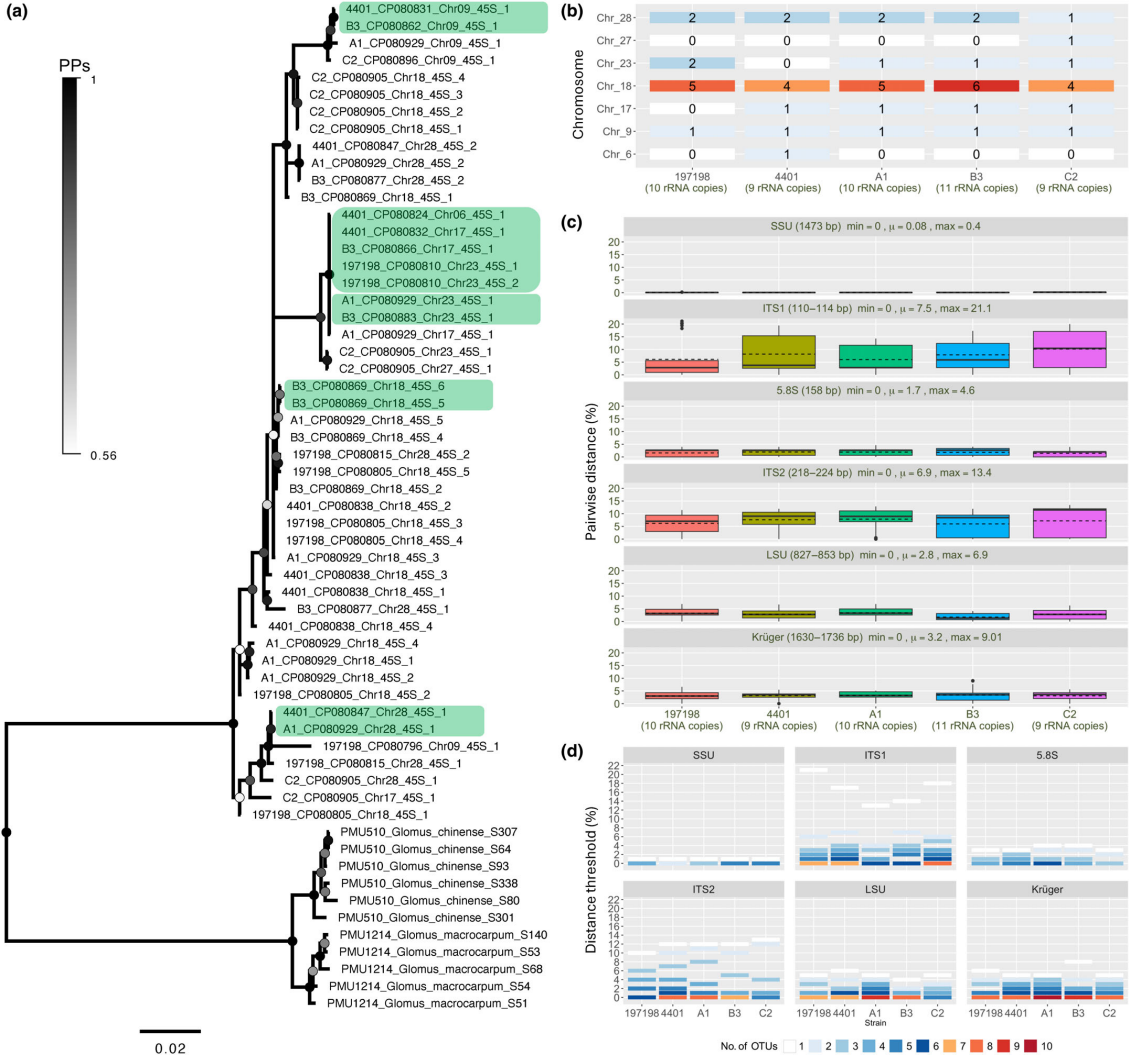
Intragenomic rDNA polymorphism across five *Rhizophagus irregularis* strains. (a) Fifty percent majority rule consensus tree derived from Bayesian analyses of the rDNA copies (2.8 kb) obtained from the whole genomes of *R. irregularis* strains sequenced in Yildirir *et al*. (2022). *Glomus chinense* and *Glomus macrocarpum* were used as outgroups. Identical sequences (0% pairwise dissimilarity) are highlighted with light green boxes. The maximum pairwise dissimilarity between the 48 rDNA copies was 4.5% (128/2808). Sequence labels indicate strain ID, GenBank accession, chromosome, and copy. Gradient colored dots on branch nodes indicate posterior probabilities (PPs), ranging from 0.5 (white) to 1 (black). The bar indicates the expected number of substitutions per site. (b) Heatmaps showing the distribution and abundance of rDNA copies on the chromosomes of *R. irregularis* strains as sequenced by Yildirir *et al*. (2022). The color intensity represents the number of copies on each chromosome. Strains are plotted on the *x*‐axis and chromosomes on the *y*‐axis. (c) Intragenomic variation (measured as percentage of pairwise distance, *y*‐axis) of rDNA copies in *R. irregularis* strains (197198, 4401, A1, B3, and C2, *x*‐axis) based on chromosome‐level genome assemblies from Yildirir *et al*. (2022). The bottom panel shows the pairwise distances calculated for a 1.5‐kb fragment spanning the region from the end of the small subunit (SSU) to the D2 region of the large subunit (LSU), targeted by the primer sets developed by Krüger *et al*. (2009). Min, μ, and max values indicate the minimum, mean, and maximum pairwise distances calculated between each rDNA copy, respectively. In the box plots, solid and dashed lines represent the median and mean values, respectively. Whiskers indicate the most extreme values within 1.5 × the interquartile range from the lower and upper quartiles. Data points beyond this range are shown as outliers. (d) Heatmap showing the number of operational taxonomic units (OTUs) based on the dissimilarity thresholds (measured as percentage of pairwise distance, *y*‐axis) used to cluster the rDNA copies of each strain. Each panel represents a different rDNA locus. One of the rDNA copies on chromosome 18 in strain B3 was excluded from the analyses because of contamination by *Daucus carota* sequences.

The 48 copies of rDNA found within the five genomes of *R. irregularis* strains (Yildirir *et al*., 2022) did not cluster by strain or chromosome, while some copies from different strains and different chromosomes were identical (Fig. 1a). The rDNA copies had an uneven distribution and variable abundance among the chromosomes, with copy numbers ranging from 9 in strains 4401 and C2 to 11 in strain B3 (Fig. 1b). Strain‐specific pairwise alignments of these copies showed average distances ranging from 0.08% (SSU) to 6.9% (ITS2), with the maximum pairwise distance of 21.1% observed for the ITS1 region in strain DAOM 197198 (Fig. 1c). For the Krüger fragment, pairwise distance was up to 9.01% in strain B3. The rDNA copies could be grouped into a single cluster for the five strains using a uniform distance threshold of 1% for the SSU region only (Fig. 1d). Clustering the SSU sequences at a distance threshold of 0% (i.e. ASV) resulted in two to five ASVs. For the other loci, sequence divergence prevented clustering into a single group using a consistent distance threshold across the five strains. Strain‐specific thresholds ranging from 13% to 21% were required to group the ITS1 sequences into a single cluster. The LSU sequences from strains DAOM 197198, A1, and C2 each clustered at a distance threshold of 5%, while the LSU sequences from strains B3 and 4401 each clustered at a distance threshold of 4% and 6%. Similarly, the sequences of the Krüger fragment grouped into a single cluster using strain‐specific distance thresholds: 4% for strains 4401, A1, C2; 6% for DAOM 197198; and 8% for B3.

**Fig. 2:**
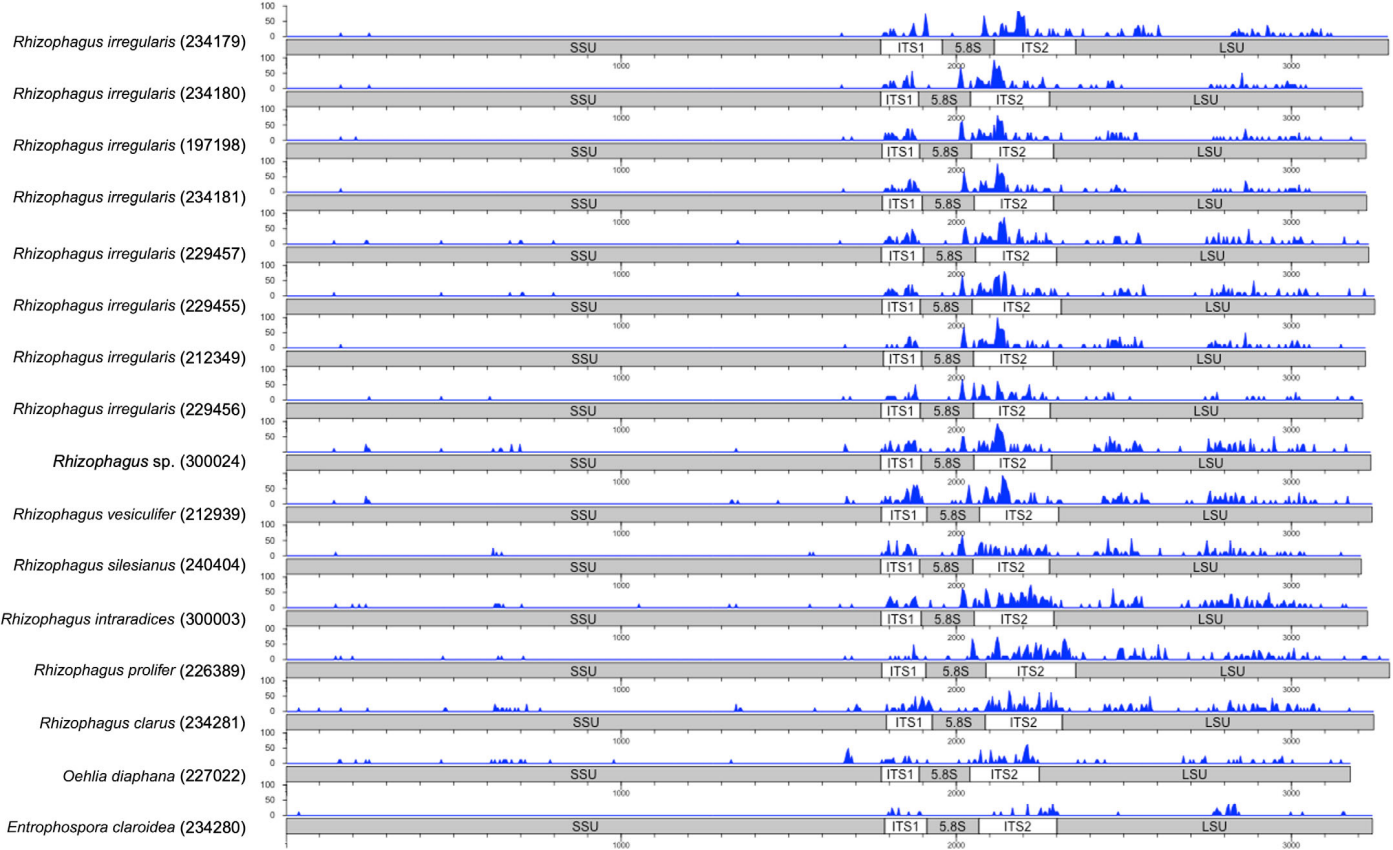
Distribution of single‐nucleotide polymorphism (SNP) density (blue peaks) across the rDNA sequences of 16 AM fungal strains cultivated under *in vitro* conditions. Culture reference identifiers (DAOMC number) are indicated in brackets. Details about cultures are provided in Supporting Information Table S1. AM, arbuscular mycorrhizal.

SNP density analysis of the rDNA loci showed that the intragenomic polymorphism of the nuclear rDNA was widespread among species within the Glomeraceae *sensu* Piroz. & Dalpé (Fig. 2). The SSU was the most conserved rDNA locus; however, SNPs were detected in different regions along its *c*. 1.8‐kb sequence. The LSU and ITS regions showed a higher SNP density in *Rhizophagus* species other than *R. irregularis*. By contrast, *Oehlia diaphana* and *Entrophospora claroidea* had significantly fewer SNPs in these regions, with 2.3‐ and 2.6‐fold fewer SNPs in the LSU region, respectively, compared with *Rhizophagus* species.

### Resolving rDNA intragenomic polymorphism with PacBio single‐spore sequencing

**Fig. 3:**
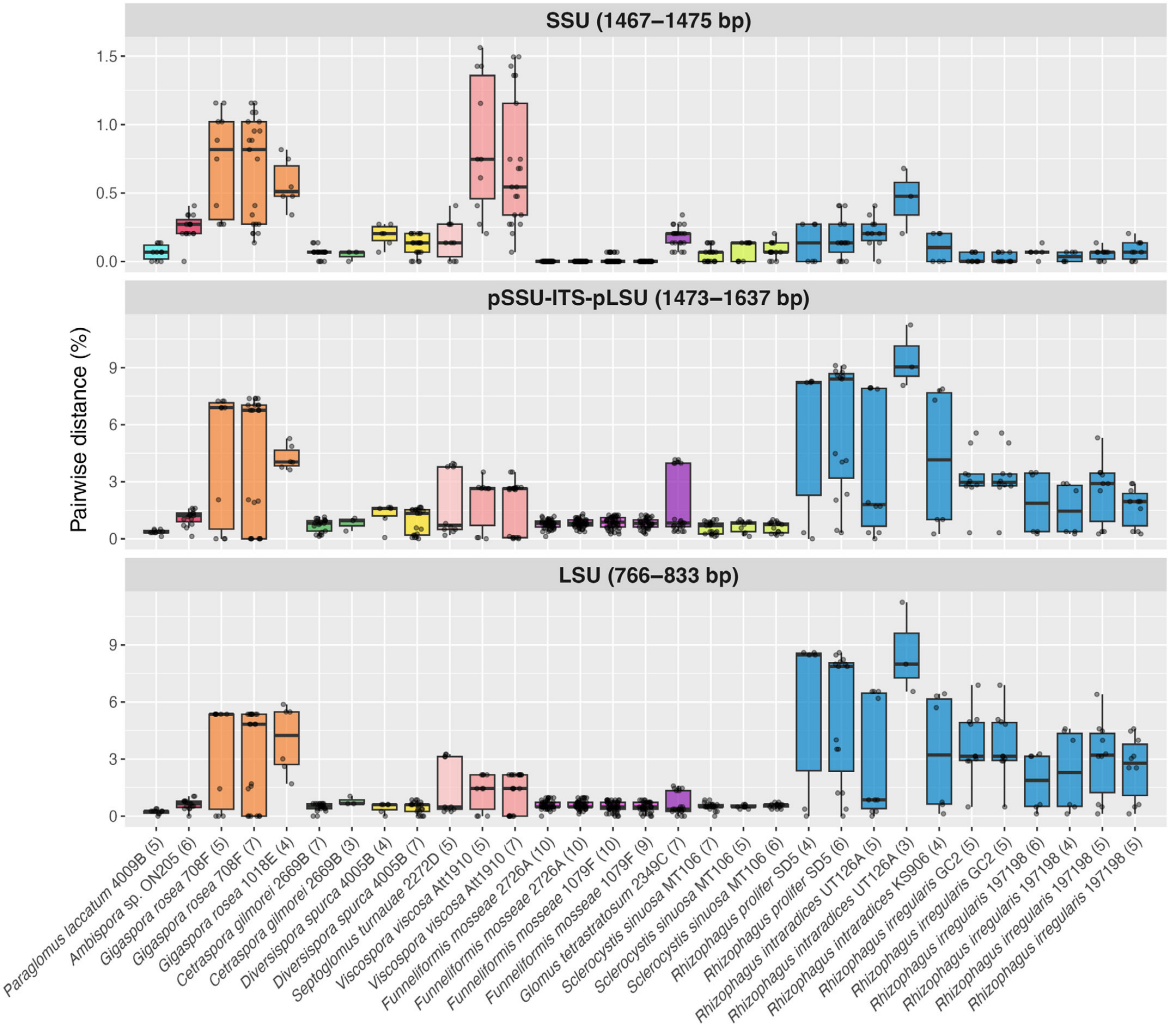
Intragenomic variation, expressed as a percentage of pairwise distance (*y*‐axis), among rDNA copies sequenced from individual spores of arbuscular mycorrhizal fungi (AMF) (*x*‐axis). Each panel corresponds to a different rDNA locus commonly targeted to profile AM fungal communities in metabarcoding studies or to describe new AM fungal species (i.e. Krüger fragment: small subunit (SSU) (*c*. 280 bp), internal transcribed spacer 1 (ITS1), 5.8S, ITS2, and large subunit (LSU) (800 bp)). Numbers in parentheses after species names and strain IDs indicate the total number of rDNA sequences included in the alignment used to calculate pairwise distances. In the box plots, solid lines represent the median values. Whiskers extend to the most extreme values within 1.5 × the interquartile range from the lower and upper quartiles. AM, arbuscular mycorrhizal.

The bioinformatic pipeline developed to analyze the PacBio HiFi reads recovered four to eight of the rDNA copies in 11 PacBio datasets of 5 *R. irregularis* strains (Tables S8, S9). Comparisons of the PacBio HiFi reads with the DAOM 197198 reference sequences (Yildirir *et al*., 2022) showed that copies of chromosomes 18, 23, and 28 were recovered, but not Chromosome 9 (Fig. S3B–D). The majority of the sequences were either identical (55% of the sequences) or different by one nucleotide (38% of the sequences) from the reference sequences. This pipeline was then used to investigate the potential intragenomic variation among rDNA copies from single spores of species from Glomerales, Archaeosporales, Diversisporales, and Paraglomerales (Fig. 3). Pairwise alignment of the rDNA copies showed that the range of intragenomic distances is uneven across AM fungal species, with variation observed between species at each rDNA locus. This is consistent with previous strain‐level observations in *R. irregularis* and confirms that a single distance threshold cannot cluster the rDNA sequences into a single OTU across AM fungal species for any locus within the rDNA operon. Even the highly conserved SSU rRNA gene showed variability, with maximum pairwise distances of 1.1% in *Gigaspora rosea* and 1.5% in *Vicospora viscosa*. Species from the genera *Cetraspora*, *Funneliformis*, and *Sclerocystis* showed the lowest intragenomic variability, with average intragenomic variability inferior to 1.3% for the Krüger fragment. The maximum intragenomic variability observed for the Krüger fragment or the LSU only ranged from 3% to 11% for species from the genera *Gigaspora* and *Rhizophagus*. *Rhizophagus prolifer* and *Rhizophagus intraradices* exhibited the highest levels of intragenomic variability, with maximum pairwise distances ranging from 7.8% to 11% for the Krüger fragment. The presence of highly divergent rDNA copies within the genomes of these two closely related species resulted in paraphyletic clades when phylogenetic relationships were inferred using either the Krüger fragment or LSU region (Fig. S4).

### Phylogenies and DNA barcoding performance of protein‐coding genes and rDNA

**Fig. 4:**
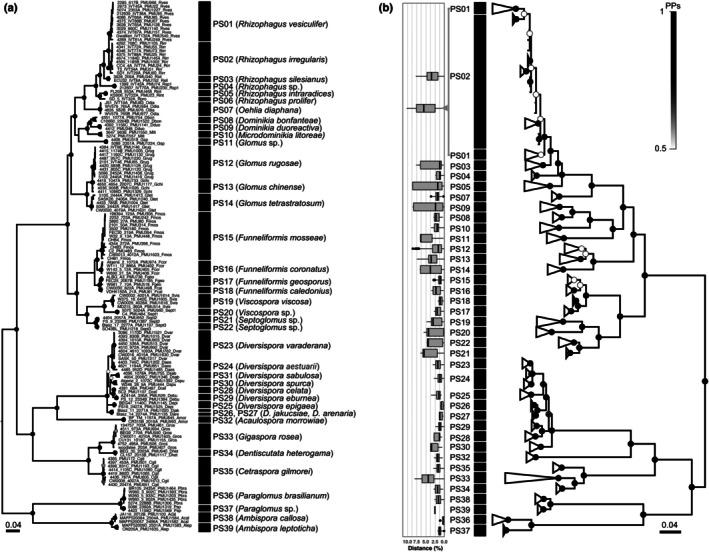
Contrasting phylogenetic signals from protein‐coding genes and rDNA. (a) Fifty percent majority rule consensus tree derived from Bayesian analyses of concatenated protein‐coding genes (glomalin, H^+^‐ATPase, RPB1) and (b) partial rDNA (small subunit (SSU), internal transcribed spacer (ITS), and long subunit (LSU)). Genomic DNA was extracted from multiple spores for each culture. Horizontal boxplots represent intraspecific pairwise distances calculated from partial rDNA sequences trimmed to correspond to the Krüger fragment (spanning from the end of the SSU to the first 800 bp of the LSU). In the box plots, solid lines represent the median values. Gradient colored dots on branch nodes indicate posterior probabilities (PPs), ranging from 0.5 (white) to 1 (black). The bar indicates the expected number of substitutions per site. In the rDNA tree, branch tips have been collapsed by phylogenetic species. Phylogenetic species (PS) identified in the concatenated phylogeny (a) are reported in the rDNA phylogeny (b), except PS06 (*Rhizophagus prolifer*) and PS31 (*Diversispora sabulosa*).

Based on the concordance of gene genealogies between protein‐coding genes and partial rDNA, 39 phylogenetic species were recognized (Table S4). The phylogenies inferred from the concatenated protein‐gene sequences (Fig. 4a) and partial rDNA (Fig. 4b) were congruent, except for *Rhizophagus vesiculifer*. Similar to the situations of *R. prolifer* and *R. intraradices* (Fig. S4), rDNA copies from *R. vesicular* and *R. irregularis* did not resolve into well‐supported monophyletic clades because of large intraspecific distances exceeding 7%.

**Fig. 5:**
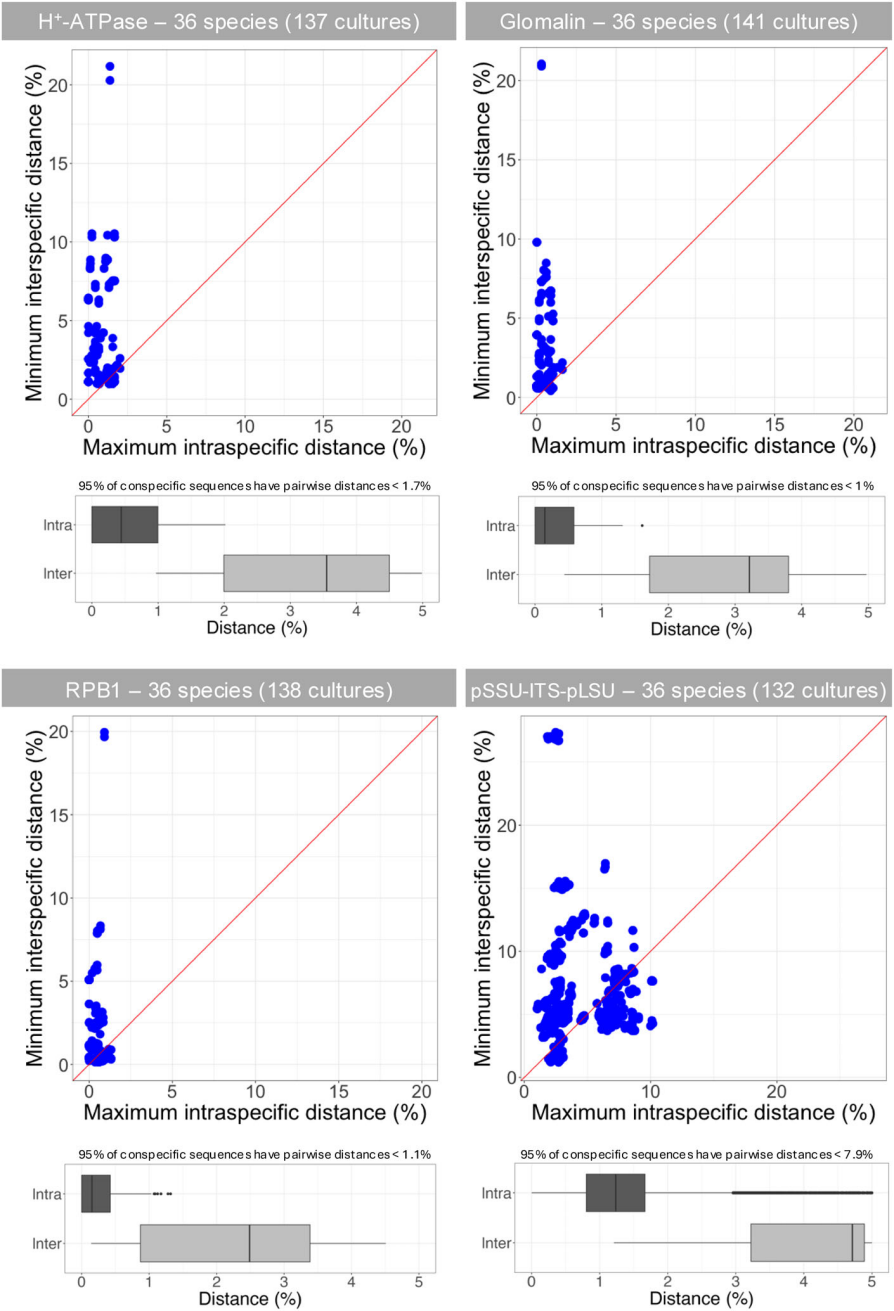
DNA barcode gap analyses. Dot plots showing the maximum intraspecific genetic distances (*x*‐axis) against the minimum interspecific distances (*y*‐axis) for each AM fungal species. Each point represents a species, with the red line indicating the function *y* = *x*. Points above the red line indicate that the corresponding species can be confidently identified by DNA barcoding. pSSU‐ITS‐pLSU represents the sequence spanning from the end of the small subunit (SSU) to the middle part of the long subunit (LSU) (1450–1650 bp, i.e. Krüger fragment). Details about cultures are provided in Supporting Information Table S3. In the box plots, solid lines represent the median values. Whiskers extend to the most extreme values within 1.5 × the interquartile range from the lower and upper quartiles. Data points beyond this range are shown as outliers. AM, arbuscular mycorrhizal; ITS, internal transcribed spacer.

The delimited phylogenetic species were then used for DNA barcoding analyses, which showed that none of the three protein‐coding genes or the partial rDNA revealed a barcode gap (Fig. 5). However, for most species analyzed, the maximum intraspecific genetic distances did not exceed the minimum interspecific distances when protein‐coding genes were sequenced: 95% of the conspecific sequences had pairwise distances of up to 1% for glomalin, 1.1% for RPB1, and 1.7% for H^+^‐ATPase, providing indicative threshold distances for deciding whether two sequences are conspecific or not. For the Krüger fragment, this threshold distance was 7.9%. Intraspecific distances for some species from the genera *Rhizophagus* (*R. irregularis* and *R. vesiculifer*) and *Glomus* (*G. chinense* and *G. rugosae*) exceeded interspecific distances with their closest relatives. Using sequences trimmed to represent the Krüger fragment, the maximum intraspecific distances were 7.7% for *R. irregularis* and 10.2% for *R. vesiculifer* (Fig. 4b), while the average interspecific distance between these two species was 6.2%. Similarly, the maximum intraspecific distances were 7.4% for *G. chinense* and 8% for *G. rugosae* (Fig. 4b), while the average interspecific distance was 5.8%. Despite this overlap between intra‐ and interspecific distances, the multigene phylogeny based on the three protein‐coding genes clearly showed these four taxa to be four distinct phylogenetic species (Fig. 4a).

## Discussion

### Nuclear rDNA intragenomic polymorphism – impact on taxonomy

Intragenomic polymorphism of AMF rDNA has been occasionally reported over the past 30 yr (e.g. Sanders *et al*., 1995; Franken & Gianinazzi‐Pearson, 1996; Lanfranco *et al*., 1999; Gamper *et al*., 2009; Thiéry *et al*., 2012, 2016; Van Kuren *et al*., 2013; House *et al*., 2016), yet ecologists and taxonomists overlooked what we show to be a core feature of AMF rDNA. Intragenomic polymorphism within the nuclear rDNA was observed in AM fungal species throughout the AM phylogenetic tree and at all rDNA loci commonly used for AM fungal community profiling and species identification. In addition, structural genomic variation was also observed as differences in rDNA copy number and chromosomal distribution; for example, 10 copies on four chromosomes in DAOM 197198, and six copies on six chromosomes in C2. The rDNA copies were not strain‐specific, indicating a weak phylogenetic signal at this level, as reported by Maeda *et al*. (2018) and Corradi *et al*. (2025). Nevertheless, the phylogenetic analysis based on partial rDNA sequences showed that a phylogenetic signal persists at the species level. Most of the phylogenetic species delimited by the concatenated protein‐coding gene phylogeny were recovered as monophyletic clades in the rDNA phylogeny.

However, the hypervariability observed in some rDNA copies at the ITS and LSU loci between closely related species introduces phylogenetic noise and blurs species delimitation when relying solely on ribosomal markers. Some rDNA copies from a given taxon may cluster into paraphyletic clades, which can be misinterpreted either as variants of a closely related species or as representing an undescribed species. This was observed in the current study with the presence of paraphyletic clades for *R. vesiculifer* and *R. prolifer –* two species well‐supported by protein‐coding genes – nested within the *R. irregularis* and *R. intraradices* clades, respectively. Similar confusing phylogenies have also been reported for other species within the genera *Rhizophagus* and *Glomus*. For example, Walker *et al*. (2021) reported that the Krüger sequences from *Rhizoglomus venetianum* were embedded within a clade of *R. irregularis* sequences, leading them to conclude that *R. venetianum* sequences may represent a subtype of *R. irregularis* rDNA variants. While this hypothesis remains plausible, the paraphyletic clades observed for *R. vesiculifer* and *R. prolifer* suggest that the conclusions drawn for *R. venetianum* should be considered with caution. The ribotypes analyzed for *R. venetianum* were obtained by Sanger sequencing of cloned amplicons and are unlikely to represent a comprehensive set of rDNA copies. Furthermore, no protein‐coding genes have been sequenced for *R. venetianum* (Turrini *et al*., 2018), preventing the assessment of its phylogenetic relationships with *R. irregularis* using an independent molecular marker. A similar case, but with an opposite conclusion, was reported in the description of *Glomus mongioiense* (Magurno *et al*., 2024). In that study, the authors identified divergent sequences in *G. chinense*, *G. ibericum*, *G. macrocarpum*, *G. mongioiense*, *G. rugosae*, and *G. tetrastratosum*, following the amplification, cloning, and Sanger sequencing of the Krüger fragment. A significant length polymorphism was observed among these species, and intraspecific sequence divergences of up to 10% were reported, introducing significant phylogenetic noise. All the species were paraphyletic when sequences with long insertions (‘L variant’ sequences) were included in the phylogenetic analyses, even when concatenated with the RPB1 sequences (see fig. S3 in Magurno *et al*., 2024). These examples highlight not only the limitations of rDNA sequences but also the risks of concatenating them with protein‐coding genes, which should instead be analyzed separately.

Another way to assign identities to AM fungal sequences is to query public databases, but querying with partial or divergent rDNA sequence can also lead to conflicting or misleading taxonomic assignments. For example, when querying GenBank using the SSU sequence of *Microdominikia litorea* (Błaszkowski *et al*., 2018), the closest match (785/787, i.e. 99.7% pairwise similarity) was ‘*Glomus indicum*’ (= *Dominikia indica* (Błaszk., Wubet & Harikumar) Błaszk., Chwat & Kovács), whereas searches using the Krüger fragment returned *M. litorea* as the best match (data not shown). Since SSU sequences of *M. litorea* are unavailable in public sequence databases, the closest match found by the Blast search is an SSU sequence of a closely related species. Similarly, when querying GenBank with the SSU sequence of *R. silesianus* (Błaszkowski *et al*., 2021), the closest match was ‘Uncultured *Glomus*’, whereas the Krüger fragment correctly returned *R. silesianus* as the best match. However, when one of the divergent rDNA copies of *R. silesianus* obtained with PacBio sequencing was used to query GenBank, the Krüger sequences misidentified as *R. intraradices* and *R. irregularis* were found as the closest matches. In this case, one of the *R. silesianus* sequences from the original species description obtained by Sanger sequencing of cloned amplicons was ranked only 26^th^, with a pairwise similarity of 94%. To improve the accuracy of AMF identification, it is essential to link the SSU with the ITS and LSU sequences used in species descriptions and characterize the rDNA variants.

### Recovering rDNA copies using PacBio sequencing

To date, the rDNA copies have only been fully characterized for *R. irregularis* (Yildirir *et al*., 2022). Until genomic data of comparable quality are available for other AMF, the PacBio sequencing and bioinformatics pipeline described in this study provides an efficient approach to recover the rDNA copies in AMF, even from single‐spore material. The pipeline includes the swarm algorithm, which uses an adaptive clustering approach rather than arbitrary clustering thresholds. It recovered partial copies of rDNA on all chromosomes, except for chromosome 9, as validated by comparison with six *R. irregularis* cultures. However, if two rDNA copies are identical in sequence, the pipeline would not distinguish them. Another limitation is illustrated by the differences in intragenomic variation observed between the two single‐spore replicates of strain UT126A in Fig. 3. In one replicate, a more divergent rDNA copy was amplified and sequenced, resulting in a higher calculated level of polymorphism. While most other replicated samples showed consistent patterns, this result underscores the potential for random amplification bias when using PacBio sequencing to recover rDNA copies from single spores.

The AML1/wLSUmBr primer set amplified 2.8‐kb‐long rDNA sequences across five AM fungal orders, encompassing the Krüger fragment used by taxonomists for species identification and description, as well as regions commonly targeted by AM fungal ecologists in high‐throughput sequencing for environmental samples (i.e. V3–V4 variable SSU regions, D1–D2 variable LSU domains). Ecological studies using high‐throughput short‐read sequencing are limited to targeting rDNA loci because universal primer sets that amplify all AM fungal lineages are currently only available for these regions. Consequently, the taxonomic assignment of short rDNA reads should be improved by sequencing using the framework described in this study and their publication in curated repositories, such as RefSeq Targeted Loci (O'Leary *et al*., 2015), Unite (Abarenkov *et al*., 2023), or EUKARYOME (Tedersoo *et al*., 2024). Accurate cataloging of intragenomic variability is essential to prevent misinterpretation of rDNA variants as distinct species and should contribute to refining global distribution, ecological, and biogeographic analyses of AMF.

### DNA barcoding AMF using protein‐coding genes vs rDNA regions

None of the three protein‐coding genes or the rDNA regions analyzed in this study contained a clear barcode gap between intra‐ and interspecific distances. Furthermore, achieving broad taxonomic coverage across the five AM fungal orders required multiple primer sets, each with order‐level specificity. Although intraspecific distances occasionally overlapped with interspecific distances for protein‐coding genes, the maximum intraspecific distance remained lower than the minimum interspecific distance for most species. This allowed the establishment of indicative thresholds for conspecific identification. However, these thresholds depend on the taxon sampling used to calculate intra‐ and interspecific distances, and they may shift as additional species and populations are analyzed (Phillips *et al*., 2022). Among the rDNA regions examined, only the SSU region showed limited intragenomic variability (maximum 1.5%), making it the most suitable target for ecological studies using high‐throughput sequencing of short reads, although it may lack resolution between closely related species (Schlaeppi *et al*., 2016).

Among the three protein‐coding genes evaluated, RPB1 (Redecker & Raab, 2006; Stockinger *et al*., 2014) is the most commonly used by AM fungal taxonomists (e.g. Błaszkowski *et al*., 2016; Symanczik *et al*., 2018; Niezgoda *et al*., 2024) as a secondary DNA barcode to complement the data from the Krüger fragment, with sequences from 82 species (34 genera, 11 families) published in GenBank. However, RPB1 includes five exons and four introns and is over 2 kb, making it too long for standard Sanger sequencing and requiring careful selection of primers (see table S1 in Stockinger *et al*., 2014). As with rDNA, it is important to target the same region within protein‐coding genes to ensure that comparable sequences are deposited in GenBank. Here, we designed two primer sets per order to amplify exons 4 and 5 of RPB1 using nested PCR. A similar approach was required for H^+^‐ATPase, for which sequences are available in GenBank for 32 species, plus 22 additional species from this study. However, paralogous copies were observed within the Gigasporaceae, making this gene a poor candidate for DNA barcoding in AMF, because gene duplication confounds species‐level identifications and phylogenetic resolution. The sequencing of glomalin also required a nested PCR approach, but the two primer sets effectively amplified species across all five orders. The primer set used for the first PCR was a degenerated version of the primers designed by Magurno *et al*. (2019). Additional primers were occasionally needed to improve Sanger sequencing for Acaulosporaceae, Entrophosporales, and Paraglomerales. To date, glomalin sequences are available in NCBI for 32 species, plus 20 additional species from this study, forming a comprehensive reference dataset. The consistent amplification of glomalin, absence of introns, and low intraspecific variability support its potential as a valuable secondary barcode for AMF.

### Conclusion

Here, we showed the extent of intragenomic rDNA polymorphism in AMF and highlighted the taxonomic pitfalls associated with the analysis of highly variable loci, particularly ITS and LSU. The high variability of these loci makes a unique genetic distance threshold unreliable for species delimitation and complicates phylogenetic inferences of closely related species, based on Krüger or LSU sequences. Sequencing protein‐coding genes is essential for reliable taxonomic identification, and taxonomists are encouraged to target the glomalin gene, together with RPB1, as secondary barcodes. Finally, we provide a molecular and bioinformatics framework for the accurate characterization of AMF and their rDNA copies, which will support the development of curated reference databases of rDNA copies from well‐characterized AMF species, an essential resource for improving sequence identification and species discovery.

## Competing interests

None declared.

## Author contributions

YD initiated and maintained the CCAMF, which provided most of the cultures used in this study. SS and CB maintained the collections and performed DNA extraction, amplification, and Sanger sequencing for the glomalin and H^+^‐ATPase genes. SS and LA performed DNA extraction and PCR for PacBio sequencing. LA and GMS performed DNA extraction, amplification, and Sanger sequencing for the H^+^‐ATPase and RPB1 genes. KD and LK performed library preparation for PacBio sequencing and PCR‐free libraries for SNP density analysis. WF and ML developed the bioinformatics pipeline under the supervision of FS. ML developed the final version of the pipeline and took care of its publication on GitHub. FS performed all analyses and writing. FS and YD received research funding for this study.

## Disclaimer

The New Phytologist Foundation remains neutral with regard to jurisdictional claims in maps and in any institutional affiliations.

## Supporting information


**Fig. S1** Workflow for amplification, PacBio sequencing, and analysis of partial rDNA from single or multiple spores of AMF.
**Fig. S2** Fifty percent majority rule consensus trees derived from Bayesian analyses of each protein‐coding gene (glomalin, H^+^‐ATPase, RPB1).
**Fig. S3** Example of the abundance distribution of the swarm clusters and IsoMDS visualizations of the relationships between selected swarm clusters from two strains of *Rhizophagus irregularis* and the 10 reference sequences of *R. irregularis* DAOM 197198.
**Fig. S4** Fifty percent majority rule consensus phylograms inferred from Bayesian analyses obtained with PacBio sequencing amplicons from individual spores of AMF.

**Table S1** List of *in vitro* cultures used for investigating SNP density (Fig. 2).
**Table S2** List of cultures used for investigating intragenomic polymorphism of rDNA in single spores through PacBio sequencing (Figs 3, S4).
**Table S3** List of cultures used for phylogenetic inferences (Figs 4, S2) and assessment of DNA barcoding performance for protein‐coding genes and rDNA (Fig. 5).
**Table S4** Summary of genealogical concordance for phylogenetic species recognition (GCPSR) among the 143 AM fungal cultures analyzed for Figs 4(a), 5, and S2.
**Table S5** Top five Blast hits from NCBI GenBank for each culture based on the protein‐coding genes glomalin, H^+^‐ATPase, and RPB1.
**Table S6** Target‐specific primers tailed with universal sequences for PacBio sequencing.
**Table S7** List of primers used for amplification and sequencing of glomalin, H^+^‐ATPase regions, and RPB1 exons 4 and 5.
**Table S8** List of cultures used to calibrate the parameters for determining the swarm cluster abundance threshold (Fig. S3).
**Table S9** Summary of *Rhizophagus irregularis* cultures sequencing data and K‐Means clustering results.
Please note: Wiley is not responsible for the content or functionality of any Supporting Information supplied by the authors. Any queries (other than missing material) should be directed to the *New Phytologist* Central Office.

## Data Availability

All data supporting the findings of this study are available in the NCBI GenBank database under the following accession nos.: glomalin sequences, PV808915–PV809055; RPB1 sequences, PV809056–PV809196; H^+^‐ATPase sequences, PV809197–PV809333; rDNA sequences used in Figs 3 and S4, PX207096–PX207274; and rDNA sequences used in Fig. 4(b), PX214440–PX215164. The complete PacBio dataset is associated with BioProject PRJNA1251049.
