# Supplementary material for: The pitfalls of rDNA‐based AMF identification: a comparative analysis of rDNA and protein‐coding genes
Source: New Phytol. 2025 Sep 12;248(3):1501–15. doi: 10.1111/nph.70557 (PMC12489285; doi:10.1111/nph.70557)
Supplement: Supplementary file 1 — Fig. S1 Workflow for amplification, PacBio sequencing, and analysis of partial rDNA from single or multiple spores of AMF. Fig. S2 Fifty percent majority rule consensus trees derived from Bayesian analyses of each protein‐coding gene (glomalin, H+‐ATPase, RPB1). Fig. S3 Example of the abundance distribution of the swarm clusters and IsoMDS visualizations of the relationships between selected swarm clusters from two strains of Rhizophagus irregularis and the 10 reference sequences of R. irregularis DAOM 197198. Fig. S4 Fifty percent majority rule consensus phylograms inferred from Bayesian analyses obtained with PacBio sequencing amplicons from individual spores of AMF. [file NPH-248-1501-s001.docx]

New Phytologist Supporting information

Article title: The pitfalls of rDNA-based AMF identification: a comparative analysis of rDNA and protein-coding genes

Authors: Franck Stefani^1^, Mario Laterrière^2^, Lobna Abdellatif^1^, Claudia Banchini^1^, Greta Mader Stevens^1^, Sylvie Séguin^1^, Kasia Dadej^1^, Lisa Koziol^1^, Wendy Findlay^1^, Yolande Dalpé^1^

Article acceptance date 12 August 2025

**Determination of the Swarm cluster abundance threshold**

Swarm v3.1.4 clusters sequences based on local, iterative growth and uses sequence abundance to delineate clusters (Mahé *et al.*, 2014, 2021). The abundance distributions of swarm clusters (i.e., dereplicated sequences with an abundance value, see **Figure S1**) were highly skewed toward low-abundance clusters while true biological sequences had higher abundances and were outliers in these distributions (**Figure S3A**). Since both the number of swarm clusters and their abundance values varied greatly between datasets, an adaptive thresholding based on k-means clustering was applied to the abundance values extracted from the sequence headers of each dataset to identify significantly abundant sequences based on their distribution. Using a seven-cluster k-means algorithm, sequences were assigned to clusters ranked by their mean abundance values. Depending on the abundance profile of the dataset, the top two or three clusters with the highest mean values were selected as significant, and the minimum abundance within these clusters was defined as the cutoff threshold. This approach allowed the identification of high-abundance sequences while filtering out low-abundance noise and accounting for dataset-specific variability. The parameters were calibrated using 11 PacBio datasets representing five *R. irregularis* strains (**Tables S8 and S9**) to retain a number of high-abundance swarm clusters not exceeding 11 (see **Figure 1B**). The similarity of these sequences was then compared with reference sequences from DAOM 197198 previously sequenced by Yildirir *et al.* (2022) (**Figure S3B, S3C, S3D)**. Sequences from the selected swarm clusters were aligned with the 10 copies of the rDNA from DAOM 197198 using MAFFT v7.49 (Katoh *et al.*, 2002; Katoh & Standley, 2013) as implemented in Geneious PrimeⓇ v2024.0 (Biomatters Ltd, Auckland, New Zealand). Pairwise genetic similarities were determined using the Hamming distance metric. Non-metric MDS was performed on the distance matrix using the *isoMDS* function (MASS package). The 10 reference sequences were used as centroids. Each swarm cluster sequence was then assigned to the nearest reference sequence (centroid) by calculating Euclidean distances. Cluster membership was determined by assigning each sequence to its nearest centroid.

Figure S1. Workflow for amplification, PacBio sequencing and analysis of partial rDNA from single or multiple spores of AMF. DNA is isolated from single or multiple spores and amplified using primer sets AML1 / wLSUmBr. After indexing the PCR products, the library is sequenced using the PacBio Sequel II SMRT sequencing technology to generate ~2.8 kb of circular consensus sequences (CCS). The CCS for each sample undergoes quality control and trimming, resulting in high quality filtered reads. These reads are then clustered using the swarm algorithm and high abundance swarm clusters are selected. The final output is a FASTA file representing all or a subset of the rDNA copies for further downstream analysis. This figure was created in BioRender (https://BioRender.com/hqsdi1a)

Figure S2. Fifty percent majority rule consensus trees derived from Bayesian analyses of each protein-coding gene (glomalin, H^+^-ATPase, RPB1). Gradient colored dots on branch nodes indicate posterior probabilities (PPs), ranging from 0.5 (white) to 1 (black). The scale bar indicates the expected number of substitutions per site. A clade was recognized as a phylogenetic species (PS) if it met either of the two GCPSR criteria: 1)

genealogical concordance: the clade was present in the majority (≥ 2 out of 3) of the single-locus phylogenies; 2) genealogical non-discordance: the clade was strongly supported in at least one single-locus phylogeny and was not contradicted in any other phylogeny at the same level of support. Light and dark green boxes indicate clades recovered as monophyletic, while orange boxes highlight clades that were non-monophyletic.

Figure S3. A) Example of the abundance distribution of the swarm clusters. The dashed red line represents the cutoff threshold for this dataset. B, C, D) isoMDS visualizations of the relationships between selected swarm clusters (colored points) from two strains of *R. irregularis* and the 10 reference sequences (black triangles) of *R. irregularis* DAOM 197198 as sequenced by Yildirir et al. (2022).

Figure S4. Fifty percent majority rule consensus phylograms inferred from Bayesian analyses obtained with PacBio sequencing amplicons from individual spores of AMF. The intragenomic variation for each species, expressed as percentage of pairwise distance among rDNA copies sequenced is shown in Figure 3. Details of the AM fungal culture analysed are provided in Table S2. Sequences related to *Rhizophagus intraradices* and *R. prolifer* are colored in blue and orange, respectively, to highlight the paraphyletic clades in phylogenies inferred using the Krüger fragment and the LSU region. Gradient colored dots on branch nodes indicate posterior probabilities (PPs), ranging from 0.5 (white) to 1 (black). The scale bar indicates the expected number of substitutions per site.

References

**Katoh K, Misawa K, Kuma K, Miyata T**. **2002**. MAFFT: a novel method for rapid multiple sequence alignment based on fast Fourier transform. *Nucleic Acids Research* **30**: 3059–3066.

**Katoh K, Standley DM**. **2013**. MAFFT multiple sequence alignment software version 7: Improvements in performance and usability. *Molecular Biology and Evolution* **30**: 772–780.

**Mahé F, Czech L, Stamatakis A, Quince C, de Vargas C, Dunthorn M, Rognes T**. **2021**. Swarm v3: towards tera-scale amplicon clustering. *Bioinformatics* **38**: 267–269.

**Mahé F, Rognes T, Quince C, de Vargas C, Dunthorn M**. **2014**. Swarm: robust and fast clustering method for amplicon-based studies. *PeerJ* **2**: e593.

**Yildirir G, Sperschneider J, Malar C M, Chen ECH, Iwasaki W, Cornell C, Corradi N. 2022.** Long reads and Hi-C sequencing illuminate the two-compartment genome of the model arbuscular mycorrhizal symbiont *Rhizophagus irregularis*. *New Phytologist* 233: 1097–1107.
